# Benthic Reef Primary Production in Response to Large Amplitude Internal Waves at the Similan Islands (Andaman Sea, Thailand)

**DOI:** 10.1371/journal.pone.0081834

**Published:** 2013-11-29

**Authors:** Carin Jantzen, Gertraud M. Schmidt, Christian Wild, Cornelia Roder, Somkiat Khokiattiwong, Claudio Richter

**Affiliations:** 1 Bentho-Pelagic Processes, Alfred Wegener Institute for Polar and Marine Research, Bremerhaven, Germany; 2 Coral Ecology, Leibniz Center for Tropical Marine Ecology, Bremen, Germany; 3 Phuket Marine Biological Centre, Phuket, Thailand; Université du Québec à Rimouski, Canada

## Abstract

Coral reefs are facing rapidly changing environments, but implications for reef ecosystem functioning and important services, such as productivity, are difficult to predict. Comparative investigations on coral reefs that are naturally exposed to differing environmental settings can provide essential information in this context. One prevalent phenomenon regularly introducing alterations in water chemistry into coral reefs are internal waves. This study therefore investigates the effect of large amplitude internal waves (LAIW) on primary productivity in coral reefs at the Similan Islands (Andaman Sea, Thailand). The LAIW-exposed west sides of the islands are subjected to sudden drops in water temperature accompanied by enhanced inorganic nutrient concentrations compared to the sheltered east. At the central island, Ko Miang, east and west reefs are only few hundred meters apart, but feature pronounced differences. On the west lower live coral cover (-38 %) coincides with higher turf algae cover (+64 %) and growth (+54 %) compared to the east side. Turf algae and the reef sand-associated microphytobenthos displayed similar chlorophyll a contents on both island sides, but under LAIW exposure, turf algae exhibited higher net photosynthesis (+23 %), whereas the microphytobenthos displayed reduced net and gross photosynthesis (-19 % and -26 %, respectively) accompanied by lower respiration (-42 %). In contrast, the predominant coral *Porites lutea* showed higher chlorophyll a tissues contents (+42 %) on the LAIW-exposed west in response to lower light availability and higher inorganic nutrient concentrations, but net photosynthesis was comparable for both sides. Turf algae were the major primary producers on the west side, whereas microphytobenthos dominated on the east. The overall primary production rate (comprising all main benthic primary producers) was similar on both island sides, which indicates high primary production variability under different environmental conditions.

## Introduction

Coral reefs are highly diverse and productive ecosystems in a nutrient poor environment (e.g. 1,2,3). This productivity can be achieved by tight coupling between organisms and functional groups and an effective recycling of essential nutrients within the reef ecosystem (e.g. 3,4,5,6,7). The primary production of coral reefs is the basis of the food web, but so far only few attempts have been conducted to quantify primary production budgets in coral reefs (e.g. 8,9,10,11). Those studies give valuable insight on coral reef ecosystems in general, but may not provide information about primary production of today’s coral reefs subjected to a changing environment ([12,13,14]). In order to evaluate these impacts, new approaches are needed to assess primary productivity of corals reefs facing changing conditions. 

The natural setting of the Similan Islands offers the unique opportunity to investigate primary production in benthic communities subjected to remarkably different environmental conditions only a few hundred meters apart. These differences are likely caused by large amplitude internal waves (LAIW) that are distributed across the Andaman Sea in the direction of the Similan Islands. Internal waves are ubiquitous in the ocean and therefore a common phenomenon, yet rarely included in ecological studies. They are mostly associated with tidal periods and moderate amplitudes ([15,16]) and form where tides interact with undersea landscapes in a density-stratified sea ([17,18]). In the Andaman Sea LAIW of great amplitude are created by tidal currents across the shallow ridges near Sumatra and the Nicobar and Andaman Islands ([19,20,21]). When LAIW reach shallower depths they transform and break delivering sub-thermocline water upslope ([22]). Comparable to pulsed upwelling in other coastal areas ([23,24,25,26]), they may deliver essential nutrients to shallow water supporting primary production in an otherwise nutrient-depleted environment. 

**Figure 1:**
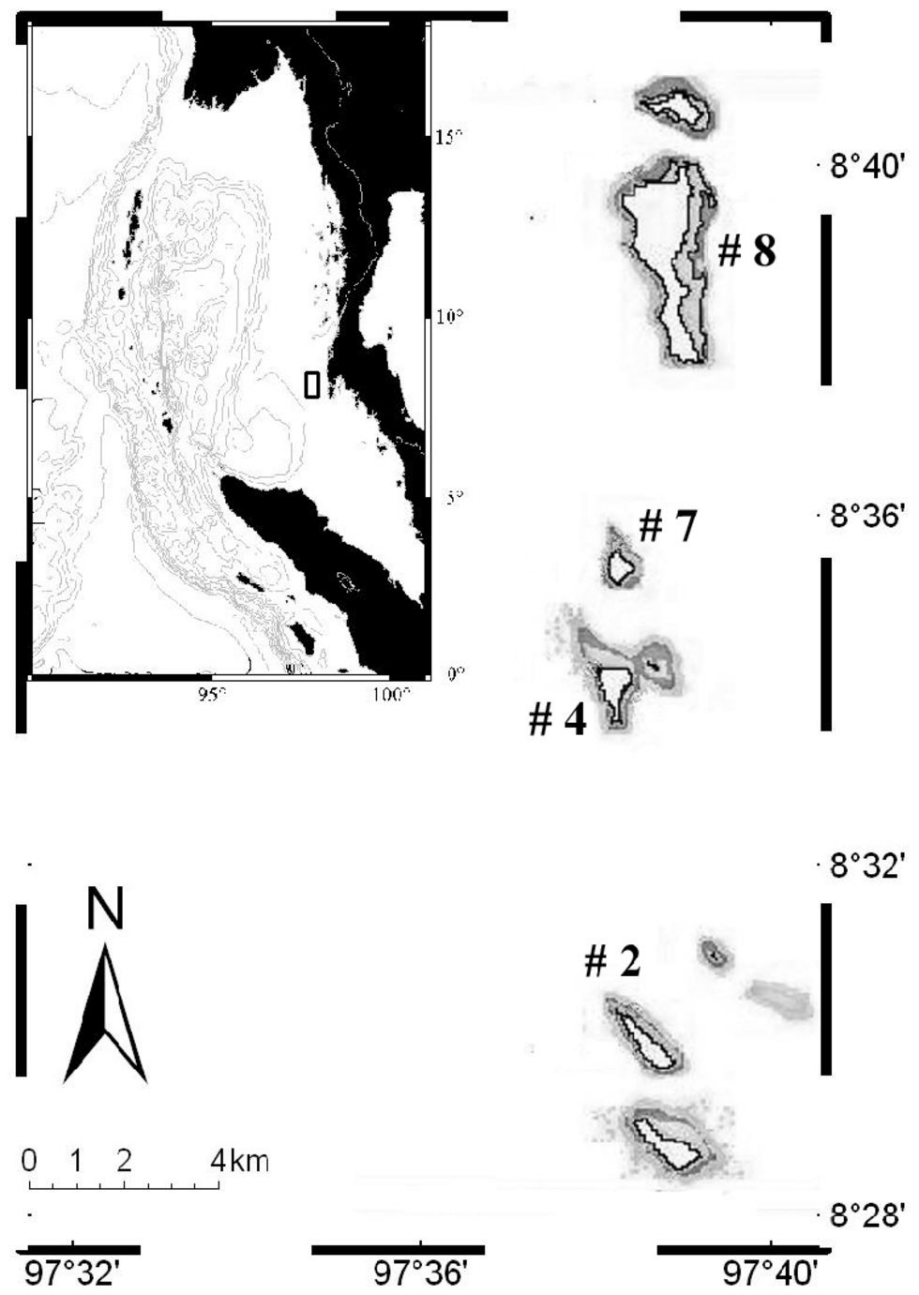
Similan Islands. Insert: location of Similan Islands (black square) within the Andaman Sea; big map: orientation of Similan island chain; Numbers of investigated islands are given (#); the principal investigation sites are at island 4 named Ko Miang.

 The Similan Island group comprises nine small islands arranged from north to south (Island # 1-9, Figure 1) located in the swash zone of LAIW ([21,22]). Here, internal wave packages are visible at the water surface as “ripplings” ([21]). Under water, LAIW approach as cold greenish water masses, causing increases in currents and pronounced decreases in temperature ([27]). Water temperature can drop down to 10° C below ambient within seconds to minutes and there is only one phenomena associated with such drops―internal waves. Temperature is negatively correlated with inorganic nutrient concentration ([27]) as LAIW transport nutrient rich and cold, deep water into shallow depths. Even though LAIW are common phenomena in coral reefs areas ([21]) their impact on benthic communities has not often been investigated ([28,27]).

On the sheltered east of the Similan Islands, where the influence of LAIW is weak, typical tropical coral reefs exist ([29]). In contrast, on the west side, where LAIW arrive directly and are much more distinct, scattered corals encrust huge granite boulders or anchor on the sand floor, but a carbonate reef framework is lacking ([30,27], www.reefbase.org/gis_maps). As primary production is generally determined by two main factors, light and inorganic nutrients, LAIW may contrarily affect primary production on the LAIW-exposed sites: higher inorganic nutrient availability may have a positive effect on photosynthetic performance, whereas lowered light levels may decrease photosynthesis. 

The various primary producing compartments or functional groups may be differently affected by incoming LAIW. The reef sands act as important biocatalytical filter systems that decompose accumulated organic material ([31,32]). Elevated concentrations of inorganic nutrients and enhanced fluxes of organic material on the west side ([28]) may impact the reef sediment’s functioning. This in turn, may affect the primary production of the microphytobenthos, which can contribute a large share to the high productivity of coral reef ecosystems ([33,34]). Turf algae are likewise important primary producers within coral reefs ([35,36]), and the enhanced inorganic nutrient availability and lower light levels on the west side may affect their growth and photosynthetic output ([37,38,26]). In contrast, corals are usually adapted to oligotrophic environments and may be at disadvantage under higher nutrient levels compared to algae ([39,40,41]). 

The aim of this study was to comparatively investigate photosynthetic performance and related characteristics such as pigment contents of dominant benthic primary producers under varying environmental settings and to explore the impact of LAIW. On the east and west side and in shallow (7 m) and deep (20 m) waters of the central Similan Island Ko Miang (Island #4) the dominant primary producers were identified as the sedimentary microphytobenthos, turf algae and scleractinian corals and their cover was quantified by line point intercept transects. Their contribution to the overall primary production was measured via photosynthesis and respiration data. Primary production budgets at each side and depth (east and west in shallow and deep water) were calculated by combining respective benthic cover and oxygen fluxes. 

Our hypothesis are as follows: we expect the LAIW to have a considerable influence on the whole coral reef ecosystem at the west side, including its primary production budget, and that this LAIW impact is clearly quantifiable via temperature drops. Further, that primary production will be influenced by adverse factors on the west side, such as less light versus higher nutrients, nevertheless we suggest that they will not rule each other out, but will shift the contribution of each primary producer, resulting in less productive corals and higher productive algae. 

## Materials and Methods

### Ethic statement

All work was conducted according to relevant national and international guidelines; therefore investigations were done with the official admission and the respective research permit of the National Research Council of Thailand in concert with the Phuket Marine Biological Centre for the Similan Islands (NRCT, permit numbers: TS0907.1/12593 and NRCT002.3/03231). We did our best to keep animal (*Porites lutea*) and algae sampling (microphytobenthos and turf algae) at a minimum and to avoid any lasting impact on the investigated reefs. As no corals were transported out of the country, no CITES permit was necessary.

### Study site and background parameters

Samplings and measurements were mainly carried out on the east (E: 8°33´57.97´´N 97°38´24.49´´E) and west (W: 8°34´15.24´´N 97°37´57.42´´E) sides of the central Similan Island # 4 (Ko Miang) from January to March 2008. Two water depths (shallow: 6-9 m; deep: 19-21 m) were selected and are referred to as sampling ‘sites’ E shallow, E deep, W shallow, W deep. A map of the Similan Islands showing their location in the Andaman Sea and the orientation of Ko Miang within the island group is given in Figure 1. Large amplitude internal waves (LAIW) occur in the Andaman Sea throughout most of the year (except August and September, [21]), and they are most pronounced at the Similan Islands from February until April ([27]). Our study was therefore conducted during LAIW high season, when they have the main impact.

Water temperature was recorded at all 4 sites using temperature (C°, TidbiT v2, Onset) loggers with a logging interval of 1 minute. Supplementary temperature loggers (TidbiT v2, Onset) with a logging interval of 4 minutes were deployed at three additional islands at 4 locations situated along the Similan Island chain (# 2, # 7, # 8 south, # 8 north, Figure 1) at the same depths and orientations as at Ko Miang (7 and 20 m, on E and W; n = 16). At all of these locations, the temperature characteristics were consistent with those at Ko Miang (same time period 02.02.-16.03.2008) and daily temperature ranges (DTR), maximum temperature – minimum temperature, provided a measure for LAIW impact at each site (Table 1 Ko Miang, Table S1 all islands). The DTR revealed increasing LAIW impact from E shallow, E deep, W shallow to W deep; therefore, sampling sites were aligned accordingly throughout this manuscript to reflect the increasing influence of LAIW. Furthermore, the consistent temperature record along the islands suggests that east (E) and west (W) faces of Ko Miang, located centrally within the Similan island chain, can be considered representative for LAIW-exposed and LAIW-sheltered island sides, respectively. 

**Table 1:** Descriptive environmental characteristics measured at Similan Island Ko Miang (Ko #4) at 4 sites (E and W, shallow = 7 m and deep = 20 m) from 02.02.2008 until 15.03.2008 in the water column and bottom sediment.

***Parameter***		**unit**	***E shallow***	***E deep***	***W shallow***	***W deep***	**R_spRsp_**	**p**
**Water column**								
**Temperature**	(mode)	(°C)	28.84	28.59	28.82	28.77		
	(mean)		28.89 ± 0.00	28.66 ± 0.00	28.75 ± 0.00	28.23 ± 0.00		
	(DTR)		1.03 ± 0.07	1.50 ± 0.10	2.44 ± 0.16	3.94 ± 0.16	0.772	**
Light	(PAR)	(µmol quanta m^-2^ s^-1^)	103.2 ± 0.48	36.2 ± 0.18	76.0 ± 0.59	23.7 ± 0.17		
Current velocity		(m s^-1^)	0.093 ± 0.005	0.075 ± 0.002	0.092 ± 0.005	0.076 ± 0.003		
Ammonium		(µM)	1.04 ± 0.09	1.19 ± 0.11	1.14 ± 0.12	1.45 ± 0.27	0.453	**
Nitrate + Nitrite			0.43 ± 0.06	0.57 ± 0.07	0.70 ± 0.14	1.19 ± 0.32	0.295	(*) 0.068
Phosphate			0.22 ± 0.02	0.25 ± 0.01	0.26 ± 0.01	0.37 ± 0.12	0.122	-
**Sediment**								
Clay and silt	(samples pooled)	%	4.33	3.85	4.03	0.29		
Fine sand			25.25	26.50	1.57	16.23		
Medium sand			42.59	46.41	47.42	35.92		
Coarse sand			18.03	20.21	41.02	33.86		
Carbonate content			86.70	83.40	72.60	78.00		
Water content			35.20	34.70	38.70	37.90		
POC content		(µg mg^-1^ sediment)	2.10 ± 0.07	2.06 ± 0.08	2.33 ± 0.07	2.21 ± 0.07	0.354	-
PN content			0.13 ± 0.01	0.06 ± 0.01	0.18 ± 0.03	0.11 ± 0.02	0.217	-
Ammonium	(pore water)	(µM)	21.20 ± 6.29	28.72 ± 7.83	25.33 ± 3.45	38.60 ± 3.69	0.655	**
Nitrate + Nitrite			2.10 ± 0.62	7.18 ± 2.10	3.70 ± 1.22	18.60 ± 3.75	0.327	-
Phosphate			8.10 ± 1.22	10.09 ± 1.37	7.79 ± 2.15	11.82 ± 1.70	0.569	*

Parameters given are water temperature (mode, mean, daily temperature range [DTR]), light conditions (photosynthetic active radiation, PAR) and current velocity in the water column, as well as grain size fractions, carbonate and water content, particulate organic carbon (POC) and particulate nitrogen (PN) of the sediment. Nutrient concentrations were determined in both water column and sediment pore water. Correlation of certain parameters to increasing LAIW impact from E shallow to W deep by Spearman rank correlation. (Rsp = Spearmen´s correlation, p = probability level, significance levels are *0.05 > P ≥ 0.01, **0.01 > P ≥ 0.001). Values are displayed as mean ± SE.

Light intensity was recorded with light (lx, Pendant, Onset) loggers at 1 minute intervals at the 4 study sites at Ko Miang and computed as daily mean around noon (11:00-14:00, 02.02.-16.03.2008). To assess the suitability of the Pendant logger data as rough proxies for the photosynthetic active radiation (PAR), a calibration experiment was carried out during 6 diurnal cycles in shallow (7 m, n = 4) and deep (20 m, n = 2) water. The light intensity (I [lx]) was measured with Pendant light loggers (as above), and photosynthetic active radiation (PAR, µmol quanta m^-2^ s^-1^) was recorded concomitantly with the internal light sensor of a submersible pulse amplitude modulated fluorometer (Diving PAM, Heinz Walz GmbH, Germany). In spite of the different optical spectra recorded by the Pendant and Diving PAM sensors, we found a fair correlation [I (lx) = 0.0101x PAR (µmol quanta m^-2^ s^-1^), R^2^ = 0.52, Figure S1]. It has to be taken into account that this is only an estimated correlation as different wave lengths penetrate increasing water depth with varying success, but in the context of this ecological study it provides sufficient resolution to compare all sites. Additional factors causing measured deviation may be a general scattering of light due to wave movements, or a diverging sensitivity of the sensors (bio fouling could be excluded due to meticulous cleaning).

Water currents were measured with 2 autonomous upward-looking Acoustic Doppler Current Profilers (ADCP, RDI Teledyne Workhorse Sentinel, 600 kHz and 300 kHz) deployed at E and W of Ko Miang in 25 m depth . The currents in the water column were measured above the transducer at 1 m vertical and 1 min temporal resolution with an accuracy of 0.3 to 0.5 % of the water velocity and a precision of ± 0.3 to 0.5 cm s^-1^. Data were imported into Matlab (RDADCP by Rich Pawlowicz, U. of British Columbia, http://www2.ocgy.ubc.ca/~rich/) and current speeds were determined by averaging the 3-D current measurements from 3 vertical bins for deep (20 m : 20 to 17 m depth) and shallow (7 m: 9 to 6 m depth). 

Water samples for inorganic nutrient analyses (ammonium, nitrate + nitrite, and phosphate) were collected during the sampling period using SCUBA (in total: E shallow, n = 11; E deep, n = 12; W shallow n = 8; W deep n = 9 samples). Water samples were filtered (GF/F filters, Ø 25 mm, nominal pore size: 0.7 µm), and the filtrate was conserved with HgCl_2_ ([42]). 

### Benthic community composition

Line point intercept transects (LPI, 50 m length, [43]), were conducted along isobaths at the E and W of Ko Miang, at 7 m and 20 m. Two (deep) to 3 (shallow) replicate transects were carried out spaced 10 m apart. Within each transect, the benthic community was sampled every 0.5 m (comprising 101 data points each). Hard substrate (live and dead coral cover and rock), sediment and turf algae cover (on all substrates and only on hard substrate), and coral morphologies (used for the 2D to 3D factors, see below) were determined. 

### Benthos Sampling and Processing

Only the top layer of the sediment was considered as photosynthetically active, as light can only penetrate a few mm into the sediment ([44]). Surface cores of 1.2 cm thickness (less was not practicable) and 1.3 cm diameter (i.e. 6.4 ml volume and 3.9 cm^2^ surface area) were taken for investigation of sediment composition and photosynthesis-related parameters. The samples were processed within 1 h after collection and used for incubation experiments (E shallow, n = 15; E deep, n = 15; W shallow, n = 14, W deep, n = 20), pigment determinations of chl-a and pheophytin content (n = 10 for each site), and analysis of sediment grain sizes (12 individual samples pooled for each site). 

Additionally cores of 5.6 cm sediment thickness (33.4 ml) were taken for pore water nutrient analysis (n = 4 for each site). Within 1 h after sampling, the cores were washed with 10 ml distilled water, centrifuged and the supernatant filtered through pre-combusted GF/F filters (pore size ~ 0.7 µm). This procedure was repeated two more times and HgCl_2_ was added to the final filtrate for conservation ([42]). Filters were frozen for later determination of particulate organic carbon (POC) and particulate total nitrogen (PN). 

Turf algae samples were defined as a conglomerate of diverse unidentified filamentous algae growing up to a height of about 1 cm. They occasionally appeared on sediment, but only the ones on hard substrate were collected for analyses: Small pieces of coral rubble and rock were collected and the associated algae removed within 1 hour after sampling using a scalpel and an airbrush filled with filtered seawater. The algae in seawater were used for incubations and later filtered (GF/F filters) and frozen for pigment analysis (E shallow, n = 5; E deep, n = 15; W shallow, n = 10; W deep, n = 15). Surface area was calculated using the best fitting geometrical form(s) of each scraped rubble piece. Only the light-exposed surfaces of the rubble fragments were considered. To determine algal growth rates, microscope slides (later referred to as algae tiles) were fixed on holders and deployed at each site. Algae tiles were sampled and displaced in a random order after two to three weeks (E shallow, n = 15; E deep, n = 15; W shallow, n = 10; W deep, n = 17). They were scraped with a scalpel and airbrushed with filtered seawater. The algae-seawater suspension was treated as above for pigment analysis. Daily growth rates were calculated as amount of chl-a (µg, see pigment determination below) per algae tile area (cm^-2^) and unit time (d^-1^). 

The massive stony coral *Porites lutea* ([45]) is the most common coral species at the Similan Islands, and one of the most abundant species in the Andaman Sea ([29]). Five colonies were sampled at each site, and two fragments were chiselled from each colony. The fragments were left at the appropriate site on racks to heal for two weeks. One replicate of each colony was used for light and the other for dark incubations, five fragments for each incubation chamber (light or dark). Coral fragments were measured with a calliper and surface areas were calculated according to Naumann et al. ([46]) with the best fitting geometric shape (half-ellipsoid). After the incubation experiments coral tissue was removed with an airbrush and filtered seawater, and homogenized with an Ultra Turrax (IKA). A subsample was filtered (GF/F filters) and frozen for pigment analysis. For the incubation procedures see below.

### Oxygen fluxes

#### Microphytobenthos and turf algae

Samples of sedimentary microphytobenthos and turf algae were incubated in Winkler bottles (~ 60 ml) with natural seawater in a water bath in a temporary laboratory. Short-term incubations (1-2 h) were conducted around noon to obtain maximum photosynthetic rates. Five independent light and dark incubations, either sediment or algae, were conducted simultaneously every day for each site (26 incubation days in total). Varying layers of net cloth were used to adjust, on a daily basis, PAR to 105 ± 16 and 39 ± 13 (µmol quanta m^-2^ s^-1^), corresponding to the average ambient east light levels at 7 m and 20 m, respectively. Light and temperature were recorded every minute by the corresponding sensor of a diving PAM and temperature loggers, respectively (see above). Incubation temperature was 31 ± 1 °C, exceeding by 1 °C the maximum temperature at E shallow *in situ*. Incubations took place under controlled conditions resulting in primary production rates independent from short-term interferences caused by the relatively unpredictable, brief temperature drops of LAIW in-situ (see above and results). 

Oxygen concentrations were determined before and after each incubation using an optode (HQ 40, Hach Lange). Repeated incubations with seawater only (i.e. controls) at simulated shallow water light conditions and in the dark (each n = 10) revealed no measurable differences in oxygen values after 2 h and were therefore not considered in the calculations.

#### Corals

A custom-built submersible flow-respirometer was used for coral incubations for 24 h at each site *in situ*. The respirometer consisted of 3 incubation chambers (light, dark and light control, each 2.1 L volume). One fragment of each sampled colony (n = 5, see above) was placed in the light chamber to measure corals’ photosynthesis during the day; the other corresponding clones were placed in the dark chamber to analyse respiration. An average of corals’ activity was therefore obtained by simultaneously incubating 5 coral fragments (dark or light). Photosynthesis and respiration were recorded in intervals during 24 h (see below) and all incubation days featured the same clear sky and calm sea, resulting in typical conditions for this time of the year. A CTD multisensor-probe (Seabird SBE 19plusV2 with an oxygen sensor and terminal 5T pump) was connected to the chambers; the set-up further contained a plumbing between valves and a programmable control unit. The unit was programmed to perform a measuring cycle as follows: (1) simultaneous flushing of chambers with ambient water, (2) consecutive measurements of initial temperature and oxygen concentrations in each of the chambers (measurements of each chamber took 40 s, chambers were always analysed in the same order), (3) 20 min incubation with intermittent stirring of chambers with external pumps (Reich submersible pump 511-0110), and (4) consecutive measurements of final temperature and oxygen concentrations in each of the chambers (intervals as initial values). Each cycle lasted 30 min and was repeated 10 to 15 times during the 24 h. 

Gross photosynthesis for each site was calculated as: net production of each cycle + respiration mean of the dark chamber, both normalized to corals’ surface area (see above). A 2^nd^ order polynomial curve showed the best fit to the data (y = ax² + bx + c); where y is the productivity [µmol O_2_ cm^-2^ h^-1^] and x is the local time [h]. A, b and c are the coefficients in the corresponding units: [µmol O_2_ cm^-2^ h^-3^], [µmol O_2_ cm^-2^ h^-2^] and [µmol O_2_ cm^-2^ h^-1^], respectively. The first derivative of time [h] is the rate [h^-1^], the second derivative is the acceleration [h^-2^] or rate change, the third is the acceleration change [h^-3^]. Light was measured concomitantly with pendant loggers (see above) during each incubation

### Sample analyses

#### Nutrients

Nutrient concentrations (water column and sedimentary pore water) were determined photometrically after Grasshof et al. ([47]) with an autoanalyser (Evolution III, Alliance Instruments, France). To relate pore water nutrient concentrations to the water volume of the small sediment cores the water content of the sediment at each site (see below) and the dilution of the pore water (due to washing processes, see above) was taken into account: nutrient concentration of the pore water (µM) = measured concentration of the sample (µM) * 30 ml aqua dest. divided by the water content of the pore water (ml). Two extreme values of pore water ammonium were removed from the data set (E deep: 109.5 µM; W shallow: 1.05 µM) according to the outlier test after the criterion of multiple standard deviations (e.g. 48).

#### Pigments

For the pigment concentration measurements, samples (either filters or sediment) were thawed, 3 ml of acetone (90%) added, shaken thoroughly, then placed in an ultrasonic bath for 15 min and stored afterwards for 24h at 4°C in the dark to extract pigments. Samples were again shaken thoroughly and then centrifuged at high speed for 15 min. The absorbance of the supernatant was measured in a photometer (Shimadzu UV 1700, 1nm slit). For sediment and turf algae chl-a and pheophytin were calculated according to Lorenzen ([49], measuring at 665 nm, before and after the acidification with HCl). For the pigment calculations of the coral tissue the equations of Jeffrey and Humphrey ([50], measuring at 750, 665, 664, 647, 630, 510 and 480 nm) were used. Pigment data were normalized by sampled sediment area, growth area of turf algae (on hard substrate) or coral surface area. 

#### Sediment characteristics

For the sediment characteristics, the grain size fraction was analysed using wet sieving (to separate the coarse fraction) and the pipette method (to separate the fine fraction; both [51]). The different grain size fractions were dried, weighed and the proportion of weight calculated. The carbonate content was determined using a Schleicher-Apparatus and precipitated lime as standard ([52]). Water content was estimated by weight difference between wet and dry sediment. Microscopic investigations on untreated sediment revealed pennate and centric diatoms as the dominating micro-algae in all sediments. Photographs of sediment surfaces from E and W are provided in Figure S2 .

Filters for POC and PN analyses were dried at 40 °C. To remove inorganic carbon HCl (1N, 100-200 µl) was added to POC filters before re-drying them. The POC and PN contents were determined using an Elemental Analyzer (NA2100 Protein) calibrated against an elemental CHNS standard (LECO).

### Primary production budget

Daily net and gross primary production (P_net_ and P_gross_) and respiration (R) of the sedimentary microphytobenthos and the turf algae were calculated using the results of the short-term incubations (maximum rates around noon and dark incubations respectively; see above). Start and end time points of photosynthetic activity by corals were obtained via the 2^nd^ order polynomial curves (see above) and used to conduct quadratic extrapolations. P_gross_ = P_net_ + R, for each incubation, with all values given in µg cm^-2^ h^-1^. For all primary producers, the integral areas of the daily curves were calculated (Mathematica 5.2, Wolfram Research, Inc. 2005), resulting in the calculated daily respiration, net and gross primary production rates. To scale daily rates to area, they were multiplied with the relative proportion of each primary producer at each site (projected area) and corrected by conversion factors (CF) between projected and actual area for turf algae and corals, respectively. For turf algae we obtained a CF value of 1.5 (i.e. a 50% increase of the actual surface relative to the projected area). For corals the *in situ* CF after Alcala and Vogt ([53]) were used (massive: 2.31, branching: 6.88, folios: 4.43). A coral 2D to 3D factor for each site was estimated using the mean of all factors for each coral growth form (branching, massive and encrusting or foliose) and weighted by the percentage cover of each coral growth form found. Gross photosynthesis is given over the period of PAR per day (12 h of sun light; see also above) and respiration for 24 h. 

### Statistical analyses

The software Statistica v 9 was used for statistical analyses. Data were tested for normal distribution and homogeneity of variances with Kolmogorov-Smirnov and Levene’s test and log-transformed if necessary. Based on the data characteristics, both, 2-factorial analysis of variance (ANOVA) with posthoc, pairwise comparisons of the adjusted group means via Tukey HSD-tests, as well as non-parametric one-factorial Kruskal-Wallis ANOVA and median test followed by multiple comparisons of mean ranks were used to test the effect of side (W and E) and depth (shallow, deep) on environmental parameters such as temperature (DTRs), light (PAR), water currents, nutrient concentrations and sediment characteristics (PN, POC), as well as on photosynthesis related characteristics (algal growth rates, pigment content, photosynthesis and respiration) of the main primary producers (microphytobenthos, algal turf, corals). Side and depth (parametric ANOVA) or site (e.g. W 7 m, non-parametric Kruskal-Wallis ANOVA) were used as treatment factors. To test whether environmental parameters or biological responses, or both, are related to DTRs (LAIW impact) the non-parametric Spearman correlation was used, setting DTRs as ordinal ranks. If not stated otherwise values are given as mean ± SE.

## Results

### Environmental background

Synchronous temperature conditions and events (temperature variations in differing strengths) each at west (W) and east (E), deep and shallow sites along the whole Similan island chain (Table S1; [27]) confirm the central Similan Island Ko Miang (Ko #4) as a representative study area for the Similan Islands. Despite the almost identical temperature mode and mean values measured at Ko Miang, daily temperature ranges (DTRs, daily maximum – minimum) were highest at W and in deep when compared to E and shallow (Table 1, ANOVA results Table S2 A). Figure S3 exemplary shows a diurnal cycle (24h, 15.02.2008) of the temperature regime at all Ko Miang sites to visualize the strengthening of temperature drops towards W and deeper waters. 

Overall current velocities were higher at W compared to E (Table 1, Kruskal Wallis test: χ^2^ = 14.23, df = 3, p < 0.03), reflecting an enhanced water flux there. The higher current speed may cause higher turbidity and accordingly light intensity as photosynthetic active radiation (PAR) was lower at W (Table 1, ANOVA results Table S2 B, Figure S4). The enhanced turbidity may also be reflected by a higher particle load as both, total particulate nitrogen (PN) and particulate organic carbon (POC), accumulated within the sediment and were higher at W than E (Table 1, ANOVA results Table S2 C, D). The sediments themselves showed deviating grain size characteristics at each site (Table 1). Almost no clay and silt was found at W deep and little fine sand for both W sites (fewest at W shallow) contrasting with finer sediments at E. More coarse sand could be found at W due to an elevated current regime. Accordingly, water content within sediments at W was higher than in ones at E. Carbonate content was one-tenth lower at W than E (Table 1). ANOVA results revealed that ammonia and phosphate concentrations did not show significant side or depth effects (Table S3 A, B) in the water column or in the sediment pore water. But pooled nitrate + nitrite concentrations revealed highest concentrations in W deep and lowest in E shallow (Table 1, ANOVA results Table S2 C). The rank correlations however, showed that ammonia and pooled nitrate + nitrite concentrations increased with greater LAIW impact (DTR), whereas phosphate concentrations failed to show any correlation (Table 1). Nutrient concentrations in sedimentary pore water revealed different characteristics. Both ammonia and phosphate concentrations were coupled to LAIW impact (DTR), whereas nitrate + nitrite concentrations did not show any correlation (Table 1). 

### Benthic community composition

**Figure 2:**
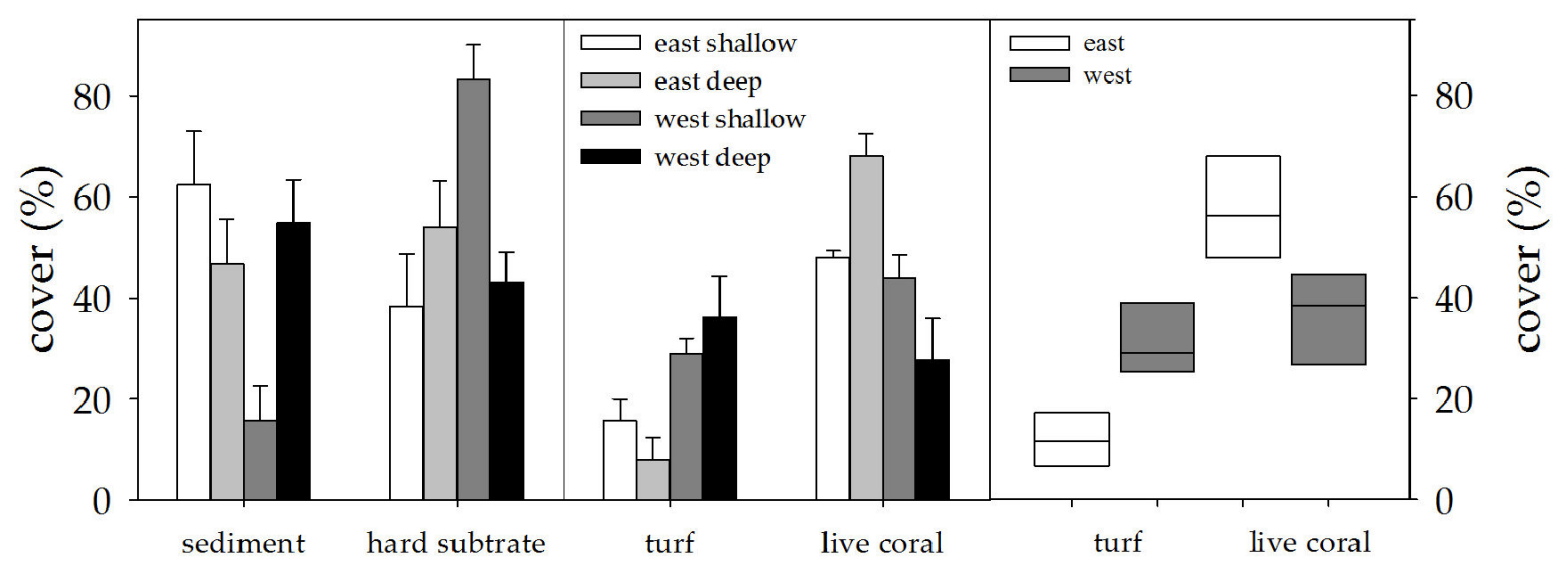
Benthic cover. Line transects on benthic cover at Ko Miang (shallow: 7m, deep: 20m) in %; live coral cover as a fraction of hard substrate; values are given as mean ± SE. The right panel shows the comparison of coral and turf algae cover between E and W to visualize the differences between sides (turf: p < 0.01, live coral: p < 0.05); values are shown as median ± 95% CI.

Benthic community composition (Figure 2, all ANOVA results: Table S4) assigned microphytobenthos (sediment associated micro-algae), turf algae and corals to be the main primary producers at Ko Miang. Sediment cover was highest at E shallow due to extensive sandy patches, medium at deep sites (E and W) and lowest at W shallow. Hard substrate (live coral cover, dead coral cover and rock) was mainly determined by the presence of rocks (granite boulders). Highest rock cover was found at W shallow, a little at W deep and none at the E sites. The W side showed higher turf algae cover on any substrate and reduced coral cover as a fraction of hard substrate especially in W deep compared to E. 

### Growth rate of turf algae and pigment content of main primary producers

Algal growth rates determined as chlorophyll-a increase (Figure S5) were higher at W and lowest at E deep (ANOVA results: Table S5). Growth rates were positively correlated to LAIW impact (DTR) (Table 2).

**Table 2:** Photosynthesis related characteristics measured at Similan Island Ko Miang (Ko #4) at 4 sites (E and W, shallow = 7 m and deep = 20 m) from 02.02.2008 until 15.03.2008 on the respective organisms of interest (turf algae, microphytobenthos in the sediment and corals).

***Compartment***	***variable***	**unit**	***E shallow***	***E deep***	***W shallow***	***W deep***	**R_sp_**	**p**
Turf algae	growth rate	(µg Chl a cm^-2^ d^-1^)	0.02 ± 0.01	0.04 ± 0.02	0.07 ± 0.03	0.05 ± 0.02	0.698	**
Sediment	chlorophyll a	(µg cm^-2^)	1.73 ± 0.59	1.73 ± 0.97	1.69 ± 1.31	1.26 ± 0.58	-0.257	0.115
Sediment	pheophytin		0.26 ± 0.24	1.19 ± 2.12	0.57 ± 0.28	0.69 ± 0.30	0.457	**
Turf algae	chlorophyll a		0.63 ± 0.29	0.76 ± 0.45	0.48 ± 0.19	0.67 ± 0.33	-0.051	0.730
Turf algae	pheophytin		0.11 ± 0.18	0.08 ± 0.09	0.10 ± 0.05	0.18 ± 0.10	0.368	*
Coral	chlorophyll a		1.67 ± 0.62	4.07 ± 1.74	3.52 ± 3.09	6.05 ± 1.85	0.657	**
Sediment	net photosynthesis	(O_2_ [µg cm^-2^ min^-1^])	0.217 ± 0.071	0.082 ± 0.074	0.176 ± 0.128	0.080 ±0.036	-0.456	**
Sediment	gross photosynthsis		0.279 ± 0.08	0.173 ± 0.110	0.212 ± 0.138	0.139 ± 0.056	-0.471	**
Sediment	respiration		-0.062 ± 0.025	-0.088 ± 0.071	-0.032 ± 0.032	-0.059 ± 0.039	0.165	0.198
Turf algae	net photosynthesis		0.046 ± 0.031	0.055 ± 0.037	0.048 ± 0.067	0.079 ± 0.053	0.275	(*) 0.078
Turf algae	gross photosynthsis		0.140 ± 0.064	0.146 ± 0.076	0.135 ± 0.080	0.123 ± 0.075	-0.084	0.596
Turf algae	respiration		-0.110 ± 0.051	-0.091 ± 0.056	-0.088 ± 0.032	-0.050 ± 0.031	0.444	**
Coral (max. rate)	net photosynthesis		0.018 ± 0.002	0.017 ± 0.001	0.024 ±0.005	0.018 ± 0.003	0.211	0.379
Coral (max. rate)	gross photosynthsis		0.028 ± 0.002	0.024 ± 0.001	0.032 ± 0.003	0.025 ± 0.003	0.01	**
Coral	respiration		-0.010 ± 0.001	-0.007 ± 0.001	-0.008 ± 0.004	-0.007 ± 0.001	0.528	*

Photosynthetic rates (net and gross) and respiration calculated via oxygen fluxes. Correlation of parameters to increasing LAIW impact from E shallow to W deep by Spearman rank correlation. (Rsp = Spearmen´s correlation, p = probability level, significance levels are *0.05 > P ≥ 0.01, **0.01 > P ≥ 0.001). Values are displayed as mean ± SE.

Pigment concentrations of turf algae on hard substrate and sediment showed similar values at all sites (ANOVA results: Table S6). Only pheophytin was positively coupled to increasing LAIW impact (DTR) (Table 2; Figure S6, right panel; Figure S7, right panel). 

Coral chlorophyll-a content exhibited lowest values at E shallow, medium values at E deep and W shallow, and highest values at W deep (Table S5, Figure S8) and was therefore higher with more pronounced DTR (Table 2). 

### Photosynthesis and respiration

**Figure 3:**
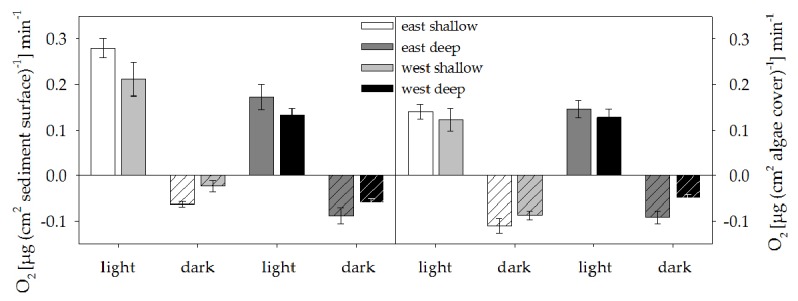
Oxygen fluxes of sediment and turf algae. Microphytobenthos and turf algae were incubated to determine oxygen fluxes (net photosynthesis and respiration); values were normalised to surface area of sediment or turf algal growth substrate (µg cm^-2^ min^-1^) and are given as means ± SE. Sedimentary net and gross photosynthesis as well as algae respiration are correlated with daily temperature ranges (DTR).

The microphytobenthos revealed net and gross photosynthetic rates negatively correlated to DTR with highest values at E shallow and lowest at W deep (Table 2). Respiration was reduced at W. Net photosynthesis and respiration were generally higher at shallow sites compared to deep ones (Figure 3, left panel; ANOVA results: Table S7).

Turf algae showed a positive coupling of net photosynthesis, but a negative correlation of respiration rates to DTR (Table 2), with respiration rates that were lower at W compared to E. Gross photosynthetic rates were constant, irrespective of side, depth or DTR (Figure 3, right panel, Table S7). 

**Figure 4:**
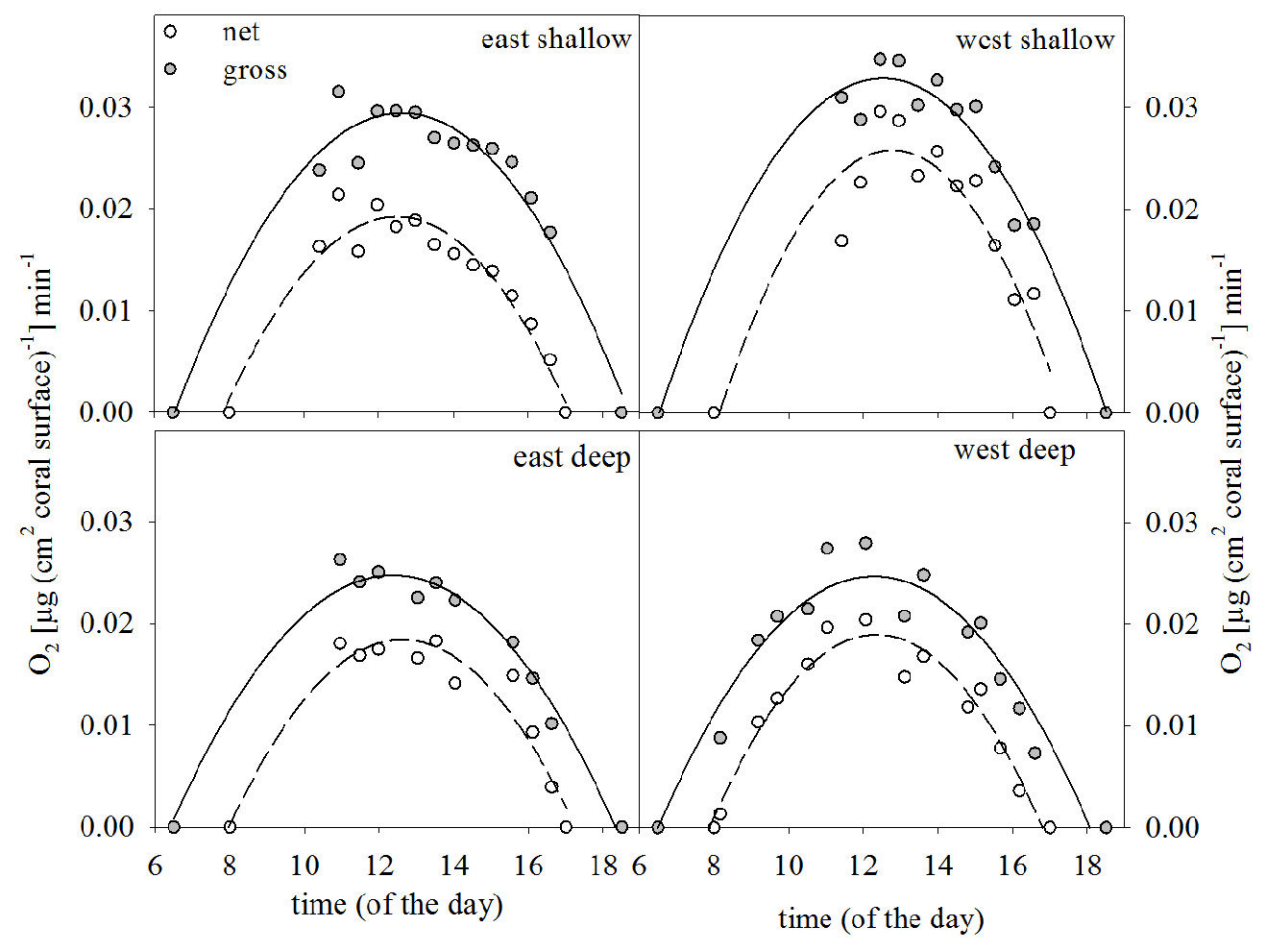
Oxygen fluxes of corals. Incubations of *P. lutea* *in* *situ* with an automated respirometer during one day at each site (shallow: 7m, deep. 20m) to determine oxygen fluxes, i.e. net and gross photosynthesis; values were normalised to surface area (µg cm^-2^). Maximum rates (at midday) of gross photosynthesis are correlated with daily temperature ranges (DTR).

Corals’ net photosynthesis over the day seemed to be relatively similar at all sites, but with more productive photosynthesis at the shallow sites (Figure 4), regardless if E or W(Table S7), despite lower light intensities at W (Table 2, Figure S4). Gross photosynthetic rates of corals around noon (net and gross, 11:00- 14:00 h) were positively correlated to DTR (Table 2), still higher in shallow. Respiration rates were similar at most sites, except for E shallow, which was double that of the other sites (Figure S9). Photosynthetic rates of corals around noon related to pigment content (chl-a^-1^) were negatively correlated to DTR, suggesting a more costly photosynthesis at W (Spearman rank correlation, p <0.001 and R_sp_ = -0.792, p <0.002 and R_sp_ = -0.745, respectively).

### Primary production budgets

Calculated net and gross primary production rates, i.e. rates attributed to photosynthetic active surface area (d^-1^), showed highest values for the microphytobenthos, followed by turf algae, whereas corals only achieved a comparatively low productivity (Table 3). The microphytobenthos showed by far the highest photosynthetic activity at E shallow, with almost 1.5-times higher photosynthetic rates than algae or corals from any other site. Taking into account the different proportions of benthic cover and the conversion factors (2D to 3D) for each site, the sediments were the major primary producers at E, especially at the shallow site (Table 3). By contrast, at W turf algae were the major primary producers. Corals contributed very little to the primary production at W deep, but a relatively large share at all other sites. 

**Table 3:** Primary production budget.

***Affiliation***	***Compartment***	***Calculated PP net (µg O_2_ cm^-2^ 12h^-1^)***	***Calculated PP gross (µg O_2_ cm^-2^ 12h^-1^)***	***3D factors***	***Cover (%)***	***PP gross (mg O_2_ m^-2^ 12h^-1^)***	***Respiration (mg O_2_ m^-2^ 24h^-1^)***
E shallow	MPB	78.1	133.8	1	59.1	791.41	-529.93
	Algae	17.8	72.0	1.5	16.6	218.89	-483.16
	Corals	7.8	13.9	4.47	18.9	118.00	-134.06
E deep	MPB	35.2	92.9	1	30.8	286.73	-427.87
	Algae	21.2	66.5	1.5	19.4	278.07	-590.56
	Corals	7.0	11.5	2.97	40.3	137.16	-120.55
W shallow	MPB	68.0	101.6	1	6.1	61.58	-19.82
	Algae	27.4	79.3	1.5	49.2	753.29	-1196.74
	Corals	10.6	15.7	3.24	26.9	133.21	-91.45
W deep	MPB	29.4	70.3	1	42.6	299.85	-372.51
	Algae	31.0	66.5	1.5	50.3	521.91	-595.14
	Corals	7.0	11.4	3.45	6.1	24.02	-21.59

Primary production budgets of microphytobenthos (MPB), turf algae (algae) and corals were estimated using actual gross photosynthetic rates (actual PP [µg oxygen cm**^*-*^** ^2^ 12h^-1^]) weighted by transect data (% cover) and conversion factors (2D to 3D, MPB: none; algae: own, see material and methods; corals: Alcala and Vogt 1997).

## Discussion

At Ko Miang, the central Similan Island, the environmental conditions differed clearly between east and west sides. Essential characteristics (biological and abiotic) were correlated to daily temperature ranges (DTR), which were in turn higher at W due to the impact of LAIW reaching the islands from W. This emphasises the relevance of LAIW, expressed as DTR, and their accompanying characteristics, such as higher nutrient concentrations and a stronger current regime, to account for the differing environmental setting at W compared to E. The coral reef communities and, in particular, the main primary producers at both island faces showed a differentiated response towards the respective prevailing abiotic conditions concerning respiration, photosynthesis and pigmentation. Nutrient concentrations were positively coupled to DTR, thus leading to higher nutrient concentrations in the water column at W of Ko Miang implying a fertilisation effect where DTR are more pronounced. Accordingly, Schmidt et al. ([27]) described a correlation of nutrient peaks with temperature drops. Turf algae seemed to benefit most from these nutrient pulses with respect to their higher growth, benthic cover and net photosynthesis on the west side. They contributed the largest part to the primary production on the LAIW-exposed west side. In contrast, the microphytobenthos (sediment associated micro-algae)―by far the most productive primary producer on the LAIW-sheltered east―had a reduced overall productivity on W. Coral (*Porites lutea*) contribution to the overall primary production was low on both sides. Photosynthetic rates at E and W were similar, but DTR associated environmental characteristics may have supported a higher investment into pigmentation on the west side. This is reflected by higher chl-a contents, especially in W deep corals, which in turn enabled them to sustain a productive photosynthetic performance on this light reduced side.

### Benthic community response

#### Sediment

The enhanced nutrient availability on W could not induce a fertilization effect on microphytobenthos’ primary production (net and gross photosynthesis), even though sediment associated microalgae are capable of an effective uptake of nutrients from pore water and water column ([54]). The microphytobenthos revealed an overall reduced productivity on the west side, regardless of similar chl-a contents on both island sides. This generally reduced sedimentary activity on W may be crucial for the overall functioning of the benthic community, as the permeable reef sediments are usually not only places of high photosynthetic productivity ([11]), but also important places for recycling of organic material ([55,31]). Roder et al. ([28]) illustrated that elevated water currents on the west side of Ko Miang provide a considerably higher plankton and POM supply to the west ecosystem compared to the one on the east. Higher concentrations of organic material in the sedimentary pore water samples from the west may therefore be caused by a higher particle load in the water column, also detectable as increased turbidity (i.e. reduced light levels). An ensuing accumulating of POM within the reef sediments may be reinforced by reduced microbial degradation ability as reflected by suppressed sedimentary respiration on W. This is further supported by higher pheophytin concentrations on the west side indicating incompletely degraded organic matter that then accumulates within the sediments. In contrast, the sedimentary pore water on W had higher inorganic nutrient concentrations (ammonium and nitrate+nitrite) when compared to E contradicting a reduced microbial degradation capability. An explanation for this discrepancy may be the augmented presence of nitrogen(N)-fixing cyanobacteria. They could raise pore water nutrient concentrations as N-fixing cyanobacteria reach high abundances under already elevated nutrient levels due to their suspended phosphor (P)-limitation ([56]). 

The sediments on the west side featured a more coarse grain size reflecting the enhanced water currents induced by incoming LAIW on W. Sedimentary grain size usually correlates negatively with organic matter content, and positively with permeability and oxygen penetration ([57,58,59,60,61]). The efficiency of permeable (reef) sediments in recycling of organic material is based on microbial diversity and abundance, which in turn is determined by sediment properties and mineralogy ([31,62]). The reduced sedimentary metabolism on the west side may therefore be partly explained by different sediment characteristics. Lower carbonate content and more coarse grain size may lead to a reduced surface area for microbial colonisation ([31,62]. This may imply consequences for the sedimentary meiofauna and the associated intensity of bioturbation ([63]), which was less pronounced on the west side (pers. observations, also visible in Figure S2).

#### Turf algae

Turf algae can be major primary producers within the benthic reef community ([64,36,11]) and their photosynthesis may be enhanced under elevated nutrient levels ([37]). Both observations could be confirmed by our study, but as net photosynthesis and respiration co-varied in response to DTR, i.e. net photosynthesis and respiration were both higher on the west side, still just not significant (p = 0.078), gross photosynthesis of turf algae remained constant, and a net increase in photosynthesis was in not detectable in the overall rate. A study by Klumpp and McKinnon ([65]) revealed an inverse relationship of turf algae biomass and gross primary productivity, possibly due to self-shading. A similar relation may apply here as turf algae obviously benefited from higher nutrient input on W in terms of enhanced growth rates, further supported by a higher observed cover, and a raised net photosynthesis, but they showed similar gross photosynthetic rates at both island sides. A dense algal turf as observed on the west side, in particular at W deep, may not be uncommon in coral reefs and can constitute up to 80 % of the total benthic cover ([65]). Turf algae growth is not only determined by nutrient availability, especially inorganic nitrogen, but also by grazing ([66,37,38]). Although grazers were present on both sides, no apparent grazing traces could be observed on any of the algae tiles (E or W). Additionally, on W, turf algae had higher growth rates, but may, to some extent, have been dying back quickly―or fading―as indicated by the high pheophytin content of algae tile samples. 

#### Corals

Corals (*Porites lutea*) displayed similar photosynthetic rates on both island sides; therefore two changes in environmental conditions―reduced light intensities and enhanced nutrient concentrations―may have compensated each other, since corals on the west side could still sustain the same photosynthetic productivity as on the east side. At W shallow, where corals showed the highest photosynthetic rates of all sites, *P. lutea* may have benefited particularly from elevated nutrient availability and relatively high light intensities compared to E shallow and W deep. Accordingly, corals at W deep exhibited the highest investment into pigmentation, facilitated by the enhanced nutrient levels ([67,68]), enabling them to use the reduced light more efficiently ([68,69,70]) and suggesting an adaptation to lower light levels. This may imply a higher energetic investment by zooxanthellae of W deep corals as increased chl-a contents are usually caused by elevated chl-a concentrations per cell and not by multiplication of zooxanthellae numbers within the host tissue. A review by Leletkin ([71]) indicates that zooxanthellae abundances should remain relatively constant within the range of nutrient concentrations and light intensities measured during our study. This is further supported by similar zooxanthellae abundances in the tissue of *P. lutea* investigated in this study irrespective of exposure or water depth (own unpublished data, two-tailed U-test, p >> 0.05). 

The relatively constant photosynthetic performance of *P. lutea* at all sites is in agreement with a study by Titlyanov ([69]), who found a stable level of corals’ primary production in a wide range of light intensities (i.e. water depths), mainly caused by reduced respiration under lower light levels. Nevertheless, *P. lutea*’s respiration rates were similar at most sites, except at E shallow, where elevated respiration rates may reflect an enhanced metabolism, due to a high-light history ([71,73]). A surplus of solar energy, which cannot be used for photochemical energy conversion, is mainly dissipated as heat, but also causes reversible photodamage. In turn, this reversible photodamage requires enzymatic reparation of photosystem II and consequently raises respiration rates ([72,74,75]).

### Primary production

Primary production budgets related to reef area for all sites were estimated by combining incubation with transect data, i.e. photosynthetic rates with benthic cover, taking into account the 2D to 3D conversion factors (Table 3). It should be noted that no light respiration could be measured. Light respiration can be considerably higher than dark respiration ([69,76]), partly due to the required reparation of reversible photodamage as mentioned above ([72,74,75]). Gross primary production may therefore be underestimated and this may explain why the overall photosynthesis - respiration ratios per site are < 1. Both sediments and algae exceeded the productivity of corals, which was mainly caused by higher absolute cover, but also to some extent by a higher photosynthetic activity. Corals contributed a rather small amount to primary production, especially at W deep. This low coral productivity is supported by a study of Rogers and Salesky ([35]) that identified turf algae as more photosynthetically productive than *Acropora palmata*. Total gross primary production budgets for each site yielded similar outputs―somewhat higher at shallow―leading to the conclusion that overall gross primary production was relatively independent of the factor side, but the contributions of the key primary producers varied under different environmental conditions. 

Although a different incubation method had to be used for corals compared to the sediment and turf algae, all measured oxygen fluxes were consistent with published rates from other reef areas (sediment: [77], [34], [31]; turf algae: [37], [78], [65]; corals: [79], [11], [69]). However, the metabolic characteristics of *P. lutea* may not be representative for scleractinian corals in general. In an earlier study comparing photosynthetic efficiency and productivity between corals ([80]), *Porites* (*P. cylindrica*) ranked among the lowest productive species; this would underestimate coral photosynthesis. *P. lutea* may also be a relatively robust species in general considering its resilience to bleaching ([81]). *In situ* huge *P. lutea* colonies were regularly observed to be covered with mucus sheets to get rid of the sedimentation, especially on the west side (personal observation), and *Porites* sp. is known to be abundant even in areas with high particle load ([82,83]). Generally, sedimentation of corals can induce a decrease in photosynthesis and respiration rates as well as a decline of several other metabolic features ([84,85]). This may cause some coral species to compete less successfully on the west side, which may lead to an overestimation of the photosynthetic performance there. 

Different current regimes on E and W may further lead to changes in photosynthetic performance *in situ*. According to Roder et al. ([28]) a higher particle load can be found on W and may be due to increased suspension. This could result in elevated nutrient and matter fluxes. Corals for instance, take up more nutrients under higher velocities ([86]) and have a higher photosynthesis ([87]). Additionally, higher currents reduce the boundary layer above the coral tissue (and above any other interface), enabling a more rapid oxygen uptake ([88]), as well as a more easy release of waste products. This could lead to a more efficient metabolism on the W side, counteracting hampering conditions such as reduced light levels or sedimentation. Overall, higher water currents on W may increase photosynthesis of the investigated primary producers *in situ* as indicated by the studies of Carpenter et al. ([78]) on turf algae, Cook and Røy ([89]) on sedimentary microphytobenthos, and Dennison and Barnes ([90]) on corals.

It should be considered that *in situ* a plenitude of microhabitats can be found, caused by the 3D structure of the reef itself or by overgrowth (or neighbouring growth) of epiphytic organisms. This effect may lead to a variety of different small-scale light and current regimes (e.g. 91,92,76). Consequently, this may cause deviations from rates (growth of turf algae and oxygen fluxes by the main primary producers) measured during this study, as our investigations could not account for these kind of variations. Incubations were obtained under relatively constant conditions, either on land (incubations in water bath) or *in situ* (algae tiles on equalling holders and incubations within an *in situ* respirometer).

### Ecological implications

The Similan Islands offer the unique opportunity to investigate coral reef ecosystem functioning and resilience in response to deviating environmental settings located in close vicinity. Our study focused on the impact of internal waves, determined as DTR, and the associated changes in water parameters on coral reef primary production. For this reason the study period was chosen when LAIW are most pronounced along the island chain ([27]). Short-lived benthic cover, e.g. turf algae cover, and metabolic performance, e.g. respiration of sediment, may change over the year according to the prevailing environmental conditions. Hence, our study may not give a general picture on the reefs’ community and metabolism throughout the year, but only of reefs impacted by short term fluctuations introduced into shallow reef areas by LAIW. 

Enhanced nutrient levels play an important role in the competition between corals and algae and may lead to a competitive advantage for algae compared to corals ([39,40,41]). Our study showed that even short-term nutrient pulses are detectable in the water column and may impact an ecosystem subjected to these changes. This may affect benthic cover as demonstrated by the enhanced turf algae cover on the west side. Furthermore, an alteration in coral reef benthic community composition―as reported from many reef locations world-wide ([39,12,13,14])―may imply changes in ecosystem-wide primary productivity or productivity in general. Accordingly, a higher growth of turf algae combined with a reduced sedimentary metabolism was detected on the west side. Our study revealed a high plasticity of the overall benthic primary production under different environmental conditions, as the overall primary production budget was similar at all sites, with only the contribution of each main primary producer varying. It should be noted that even though the overall budget remained similar, functional groups (e.g. the sediments on the west side) may show a depressed metabolism and therefore an altered contribution to reef functioning. Further investigations are needed, particularly focusing on changing coral reef ecosystems and their performances including coherences in the trophic net.

## Supporting Information

Figure S1 Light intensity (lx) to photosynthetic active radiation (PAR, µmol quanta m^-2^ s-^1^) correlation was obtained by comparing data of pendant loggers and the light sensor of a diving PAM during six days (4 at 7m and 2 at 20m water depth, respectively); R^2^: 0.52; I (lx) = 0.0101 * PAR (µmol quanta m^-2^ s-^1^).
(EPS)

Figure S2 **Visual sediment characteristics.** Exemplary photographs of sediment surfaces at the sites of Ko Miang (E and W; shallow: 7m, deep: 20m, ~ 30 cm above bottom); higher bio-turbation on the east and more coarse grain size on the west.
(EPS)

Figure S3 **Temperature regime at sample sites of Ko Miang (shallow: 7m, deep: 20m); one day is exemplarily plotted (24h, 15.02. 2008) to show the varying impact of large amplitude internal waves (LAIW), i.e. short-term temperature drops.**
(EPS)

Figure S4 **Diurnal cycles of PAR.** Ambient light was recorded during *in* *situ* incubations of *P. lutea* at each of the incubation days. Light intensity (lx) was converted into PAR (photosynthetic active radiation [µmol quanta m^-2^ s^-1^]) using the correlation I (lx) = 0.0101 * PAR (µmol quanta m^-2^ s-^1^); see also Figure S1, shallow: 7m, deep: 20m. The different shapes of the respective E and W diurnal light courses are due to differential shadowing by the island in the morning (W) and evening (E). Both opposing study sides had a similar distance from shore and as the island is symmetrically shaped, thus resulting in a mirrored shift, i.e. at morning and evening of the diurnal light courses, respectively.
(EPS)

Figure S5 **Growth rates of turf algae.** Microscopic slides (algae tiles) were deployed *in* *situ* at the sample sites of Ko Miang and displaced in random order after 1-3 weeks (shallow: 7m, deep: 20m; E shallow, n: 15; E deep, n: 15; W shallow, n: 10; W deep, n: 17); algal growth was determined as chl a content (µg cm^-2^ d^-1^; median ± 95% CI); algal growth rates are correlated to daily temperature ranges (DTR) of the respective sites (see also Table 2).
(EPS)

Figure S6 **Pigment content of sediment.** Chl-a and Pheophytin content of sediment from sample sites of Ko Miang (E and W, shallow: 7m, deep: 20m), normalised to sediment surface area (µg cm^-2^; median ± 95% CI); sedimentary pheophytin content is correlated to daily temperature ranges (DTR) of the respective sites (see also Table 2).
(EPS)

Figure S7 **Pigment content of turf algae.** Chl a and pheophytin content of turf algae from the sites at Ko Miang (shallow: 7m, deep. 20m) normalised to growth area (µg [cm^2^ hard substrate]^-1^, median ± 95% CI); algal pheophytin content correlated to daily temperature ranges (DTR) of the respective sites (both see also Table 2).
(EPS)

Figure S8 **Pigment content of corals.** Chl-a content of *P. lutea* from sample sites at Ko Miang (E and W, shallow: 7m, deep: 20m) normalised to coral surface area (µg cm^-2^, median ± 95% CI); pigment content is correlated with daily temperature ranges (DTR, see also Table 2).
(EPS)

Figure S9 **Respiration of corals.** Incubations of *P. lutea* *in* *situ* with an automated respirometer during one day at each site (E and W, shallow: 7m, deep: 20m) of Ko Miang to determine oxygen fluxes, i.e. respiration; values were normalised to surface area (µg cm^-2^) and are given as means ± SE.
(EPS)

Table S1 **Daily temperature ranges (DTRs as max – min) at 4 of the Similan Islands (# 4, #2, #7, #8 south, # 8 north), each at 4 sites: E shallow, E deep, W shallow, W deep (shallow: 7m, deep: 20m); recording time 02.02.** -16.03.2008; for island orientation please see also Figure 1; values are given as mean ± SE; n.a. values were not available.
(DOC)

Table S2 **Analysis of variance (2-factorial ANOVA) for (**A**) daily temperature ranges (DTRs), (**B**) mean light conditions (photosynthetic active radiation [PAR, µmol photons m-2 s-1], measured between 11 am and 2 pm), and sediment content of (**C**) particulate nitrogen (PN) and (**D**) particulate organic carbon (POC) (µg mg-1).** All parameters measured at Similan Island Ko Miang (Ko #4) at 4 sites (E and W, shallow and deep) from 02.02.2008 until 15.03.2008. Side (W, E) and depth (shallow = 7 m and deep = 20 m) as treatment factors, posthoc pair wise comparisons of group means via Tukey HSD-tests (df = degrees of freedom; MS = means square; F = F-value; p = probability level, significance levels are *0.05 > P ≥ 0.01, **0.01 > P ≥ 0.01, ***P < 0.001).
(DOC)

Table S3 **Analysis of variance (2-factorial ANOVA) for nutrient concentrations (**A**) phosphate, (**B**) ammonium, and (**C**) pooled nitrate +nitrite (µM) in pore water and water column.** Samples were taken at all sites of at Similan Island Ko Miang (Ko #4) (E and W, shallow = 7 m and deep = 20 m) between 02.02.2008 and 15.03.2008. Side (W, E) and depth (shallow and deep) as treatment factors, posthoc pair wise comparisons of the group means via Tukey HSD-tests (df = degrees of freedom; MS = means square; F = F-value; p = probability level, significance levels are *0.05 > P ≥ 0.01, **0.01 > P ≥ 0.01, ***P < 0.001).
(DOC)

Table S4 **Analysis of variance (2-factorial ANOVA) for benthic cover of sediment, hard substrate (live and dead coral and rock), algal turf and live coral.** Cover data from line transects at all sites at Similan Island Ko Miang (Ko #4; E and W, shallow = 7 m and deep = 20 m). Side (W, E) and depth (shallow and deep) as treatment factors, posthoc pair wise comparisons of the group means via Tukey HSD-tests (df = degrees of freedom; MS = means square; F = F-value; p = probability level, significance levels are are *0.05 > P ≥ 0.01, **0.01 > P ≥ 0.001, ***P < 0.001).
(DOC)

Table S5 **Analysis of variance (2-factorial ANOVA) for growth rates of turf algae (µg Chla cm-2 d-1) and chlorophyll a per coral area (µg cm-2).** Both parameters measured on samples from all sites at Similan Island Ko Miang (Ko #4; E and W, shallow = 7 m and deep = 20 m) between 02.02.2008 and 15.03.2008 (F = F-value; p = probability level, significance levels are **0.01 > P ≥ 0.001, ***P < 0.001).
(DOC)

Table S6 **Analysis of variance (2-factorial ANOVA) for pigment content of (**A**) turf algae and (**B**) sediment (µg cm-2).** Both parameters measured on samples from all sites at Similan Island Ko Miang (Ko #4; E and W, shallow = 7 m and deep = 20 m) between 02.02.2008 and 15.03.2008. Side (W, E) and depth (shallow and deep) as treatment factors, posthoc pair wise comparisons of the group means via Tukey HSD-tests (df = degrees of freedom; MS = means square; F = F-value; p = probability level, significance level is ***P < 0.001).
(DOC)

Table S7 **Analysis of variance (2-factorial ANOVA) for photosynthesis (net and gross) and respiration of (**A**) microphytobenthos (sediment), (**B**) algal turf, and (**C**) corals (O2 [µg cm-2 min-1]).** All rates measured on samples from all sites at Similan Island Ko Miang (Ko #4; E and W, shallow = 7 m and deep = 20 m) between 02.02.2008 and 15.03.2008. Side (W, E) and depth (shallow and deep) as treatment factors, posthoc pair wise comparisons of the group means via Tukey HSD-tests (df = degrees of freedom; MS = means square; F = F-value; p = probability level, significance levels are *0.05 > P ≥ 0.01, **0.01 > P ≥ 0.001, ***P < 0.001).
(DOC)
