# Supplementary material for: Benthic Reef Primary Production in Response to Large Amplitude Internal Waves at the Similan Islands (Andaman Sea, Thailand)
Source: PLoS One. 2013 Nov 29;8(11):e81834. doi: 10.1371/journal.pone.0081834 (PMC3843706; doi:10.1371/journal.pone.0081834)
Supplement: Table S1 — Daily temperature ranges (DTRs as max – min) at 4 of the Similan Islands (# 4, #2, #7, #8 south, # 8 north), each at 4 sites: E shallow, E deep, W shallow, W deep (shallow: 7m, deep: 20m); recording time 02.02. -16.03.2008; for island orientation please see also Figure 1; values are given as mean ± SE; n.a. values were not available. (DOC) [file pone.0081834.s010.doc]

**Table S1** Daily temperature ranges (DTRs as max – min) at 4 of the Similan Islands (# 4, #2, #7, #8 south, # 8 north), each at 4 sites: E shallow, E deep, W shallow, W deep (shallow: 7m, deep: 20m); recording time 02.02.-16.03.2008; for island orientation please see also Figure 1; values are given as mean ± SE; n.a. values were not available.
