# Supplementary material for: Benthic Reef Primary Production in Response to Large Amplitude Internal Waves at the Similan Islands (Andaman Sea, Thailand)
Source: PLoS One. 2013 Nov 29;8(11):e81834. doi: 10.1371/journal.pone.0081834 (PMC3843706; doi:10.1371/journal.pone.0081834)
Supplement: Table S2 — Analysis of variance (2-factorial ANOVA) for (A) daily temperature ranges (DTRs), (B) mean light conditions (photosynthetic active radiation [PAR, µmol photons m-2 s-1], measured between 11 am and 2 pm), and sediment content of (C) particulate nitrogen (PN) and (D) particulate organic carbon (POC) (µg mg-1). All parameters measured at Similan Island Ko Miang (Ko #4) at 4 sites (E and W, shallow and deep) from 02.02.2008 until 15.03.2008. Side (W, E) and depth (shallow = 7 m and deep = 20 m) as treatment factors, posthoc pair wise comparisons of group means via Tukey HSD-tests (df = degrees of freedom; MS = means square; F = F-value; p = probability level, significance levels are *0.05 > P ≥ 0.01, **0.01 > P ≥ 0.01, ***P < 0.001). (DOC) [file pone.0081834.s011.doc]

**Table S2** Analysis of variance (2-factorial ANOVA) for (A) daily temperature ranges (DTRs), (B) mean light conditions (photosynthetic active radiation [PAR, µmol photons m-2 s-1], measured between 11 am and 2 pm), and sediment content of (C) particulate nitrogen (PN) and (D) particulate organic carbon (POC) (µg mg-1). All parameters measured at Similan Island Ko Miang (Ko #4) at 4 sites (E and W, shallow and deep) from 02.02.2008 until 15.03.2008. Side (W, E) and depth (shallow = 7 m and deep = 20 m) as treatment factors, posthoc pair wise comparisons of group means via Tukey HSD-tests (df = degrees of freedom; MS = means square; F = F-value; p = probability level, significance levels are *0.05 > P ≥ 0.01, **0.01 > P ≥ 0.01, ***P < 0.001).
