# Supplementary material for: Benthic Reef Primary Production in Response to Large Amplitude Internal Waves at the Similan Islands (Andaman Sea, Thailand)
Source: PLoS One. 2013 Nov 29;8(11):e81834. doi: 10.1371/journal.pone.0081834 (PMC3843706; doi:10.1371/journal.pone.0081834)
Supplement: Table S4 — Analysis of variance (2-factorial ANOVA) for benthic cover of sediment, hard substrate (live and dead coral and rock), algal turf and live coral. Cover data from line transects at all sites at Similan Island Ko Miang (Ko #4; E and W, shallow = 7 m and deep = 20 m). Side (W, E) and depth (shallow and deep) as treatment factors, posthoc pair wise comparisons of the group means via Tukey HSD-tests (df = degrees of freedom; MS = means square; F = F-value; p = probability level, significance levels are are *0.05 > P ≥ 0.01, **0.01 > P ≥ 0.001, ***P < 0.001). (DOC) [file pone.0081834.s013.doc]

**Table S4** Analysis of variance (2-factorial ANOVA) for benthic cover of sediment, hard substrate (live and dead coral and rock), algal turf and live coral. Cover data from line transects at all sites at Similan Island Ko Miang (Ko #4; E and W, shallow = 7 m and deep = 20 m). Side (W, E) and depth (shallow and deep) as treatment factors, posthoc pair wise comparisons of the group means via Tukey HSD-tests (df = degrees of freedom; MS = means square; F = F-value; p = probability level, significance levels are are *0.05 > P ≥ 0.01, **0.01 > P ≥ 0.001, ***P < 0.001).
