# Supplementary material for: Benthic Reef Primary Production in Response to Large Amplitude Internal Waves at the Similan Islands (Andaman Sea, Thailand)
Source: PLoS One. 2013 Nov 29;8(11):e81834. doi: 10.1371/journal.pone.0081834 (PMC3843706; doi:10.1371/journal.pone.0081834)
Supplement: Table S5 — Analysis of variance (2-factorial ANOVA) for growth rates of turf algae (µg Chla cm-2 d-1) and chlorophyll a per coral area (µg cm-2). Both parameters measured on samples from all sites at Similan Island Ko Miang (Ko #4; E and W, shallow = 7 m and deep = 20 m) between 02.02.2008 and 15.03.2008 (F = F-value; p = probability level, significance levels are **0.01 > P ≥ 0.001, ***P < 0.001). (DOC) [file pone.0081834.s014.doc]

**Table S5** Analysis of variance (2-factorial ANOVA) for growth rates of turf algae (µg Chla cm-2 d-1) and chlorophyll a per coral area (µg cm-2). Both parameters measured on samples from all sites at Similan Island Ko Miang (Ko #4; E and W, shallow = 7 m and deep = 20 m) between 02.02.2008 and 15.03.2008 (F = F-value; p = probability level, significance levels are **0.01 > P ≥ 0.001, ***P < 0.001).
