# Supplementary material for: Benthic Reef Primary Production in Response to Large Amplitude Internal Waves at the Similan Islands (Andaman Sea, Thailand)
Source: PLoS One. 2013 Nov 29;8(11):e81834. doi: 10.1371/journal.pone.0081834 (PMC3843706; doi:10.1371/journal.pone.0081834)
Supplement: Table S6 — Analysis of variance (2-factorial ANOVA) for pigment content of (A) turf algae and (B) sediment (µg cm-2). Both parameters measured on samples from all sites at Similan Island Ko Miang (Ko #4; E and W, shallow = 7 m and deep = 20 m) between 02.02.2008 and 15.03.2008. Side (W, E) and depth (shallow and deep) as treatment factors, posthoc pair wise comparisons of the group means via Tukey HSD-tests (df = degrees of freedom; MS = means square; F = F-value; p = probability level, significance level is ***P < 0.001). (DOC) [file pone.0081834.s015.doc]

**Table S6** Analysis of variance (2-factorial ANOVA) for pigment content of (A) turf algae and (B) sediment (µg cm-2). Both parameters measured on samples from all sites at Similan Island Ko Miang (Ko #4; E and W, shallow = 7 m and deep = 20 m) between 02.02.2008 and 15.03.2008. Side (W, E) and depth (shallow and deep) as treatment factors, posthoc pair wise comparisons of the group means via Tukey HSD-tests (df = degrees of freedom; MS = means square; F = F-value; p = probability level, significance level is ***P < 0.001).
