# Supplementary material for: Benthic Reef Primary Production in Response to Large Amplitude Internal Waves at the Similan Islands (Andaman Sea, Thailand)
Source: PLoS One. 2013 Nov 29;8(11):e81834. doi: 10.1371/journal.pone.0081834 (PMC3843706; doi:10.1371/journal.pone.0081834)
Supplement: Table S7 — Analysis of variance (2-factorial ANOVA) for photosynthesis (net and gross) and respiration of (A) microphytobenthos (sediment), (B) algal turf, and (C) corals (O2 [µg cm-2 min-1]). All rates measured on samples from all sites at Similan Island Ko Miang (Ko #4; E and W, shallow = 7 m and deep = 20 m) between 02.02.2008 and 15.03.2008. Side (W, E) and depth (shallow and deep) as treatment factors, posthoc pair wise comparisons of the group means via Tukey HSD-tests (df = degrees of freedom; MS = means square; F = F-value; p = probability level, significance levels are *0.05 > P ≥ 0.01, **0.01 > P ≥ 0.001, ***P < 0.001). (DOC) [file pone.0081834.s016.doc]

**Table S7** Analysis of variance (2-factorial ANOVA) for photosynthesis (net and gross) and respiration of (A) microphytobenthos (sediment), (B) algal turf, and (C) corals (O2 [µg cm-2 min-1]). All rates measured on samples from all sites at Similan Island Ko Miang (Ko #4; E and W, shallow = 7 m and deep = 20 m) between 02.02.2008 and 15.03.2008. Side (W, E) and depth (shallow and deep) as treatment factors, posthoc pair wise comparisons of the group means via Tukey HSD-tests (df = degrees of freedom; MS = means square; F = F-value; p = probability level, significance levels are *0.05 > P ≥ 0.01, **0.01 > P ≥ 0.001, ***P < 0.001).
